# Advanced Biomaterial for Dual‐Drug Release: A Hydrogel‐Microparticle Approach

**DOI:** 10.1002/bip.70049

**Published:** 2025-09-13

**Authors:** Jose Gregorio Fontainez Garrido, Newton Andreo Filho, Fabiana Perrechil, Mariana Agostini de Moraes

**Affiliations:** ^1^ Department of Chemical Engineering Federal University of São Paulo—UNIFESP Diadema Brazil; ^2^ Department of Pharmaceutical Science Federal University of São Paulo—UNIFESP Diadema Brazil; ^3^ School of Chemical Engineering University of Campinas—UNICAMP Campinas Brazil

**Keywords:** biopolymers, controlled release, dual‐drug delivery, hydrogel, microparticles, polysaccharides, wound healing

## Abstract

Advanced biomaterials with dual drug delivery represent a promising strategy to enhance therapeutic outcomes in wound treatment. This work aimed to combine antimicrobial and analgesic actions in a single platform, enabling the simultaneous release of both drugs from an advanced dual‐drug delivery system based on a combined hydrogel and microparticle approach. The system was composed of alginate microparticles containing the antibiotic gentamicin incorporated into a gellan gum/collagen hydrogel matrix, in which the local anesthetic bupivacaine was directly loaded. The resulting composite was thoroughly characterized in terms of its morphological, physicochemical, mechanical, rheological, and thermal properties, as well as drug release profiles. The incorporation of microparticles significantly influenced the structural and functional behavior of the hydrogel, particularly at higher microparticle concentrations (50% w/v). Notably, the microparticles played a crucial role in maintaining the hydrogel's integrity in the presence of both drugs and enabled their controlled and simultaneous release, with each exhibiting distinct release kinetics. These findings highlight the potential of this hydrogel and microparticle composite as an advanced material for wound dressings, capable of promoting healing while simultaneously providing localized pain relief.

## Graphical abstract

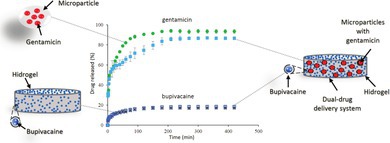

## 1 Introduction

Wound healing presents significant challenges for patients with chronic diseases like diabetes mellitus, leading to heightened risks of infection and amputation [1, 2]. Furthermore, this condition can cause ongoing pain, adversely affecting patients' quality of life and their daily activities [3]. These complications impact millions worldwide, with projections indicating that by 2030, there will be 439 million individuals living with diabetes, and around 20% of them will experience poor wound healing [4]. Dual‐drug delivery systems are a promising strategy for improving diabetic wound repair [5]. These systems have garnered significant interest in optimizing therapeutic effects by combining different drugs [5, 6, 7], allowing for simultaneous release without interaction between the drugs [8]. This approach enables the administration of lower drug doses compared to monotherapy, thereby reducing side effects and toxicity associated with higher doses. Additionally, dual‐drug delivery systems can contribute to drugs reaching the site of action and maintain sufficient concentrations for an appropriate duration, improving overall efficacy. However, a key challenge in this area is achieving independent control over the release of each drug, ensuring that each agent acts at specific stages of treatment [9, 10, 11, 12].

Various systems can be employed for dual‐drug delivery, including hydrogels, microparticles, and their combinations. One example is a system consisting of microparticles embedded in a hydrogel, where one drug is encapsulated within the microparticles and the other is incorporated in the hydrogel [13]. When considering materials for dual‐drug delivery systems, biopolymers stand out due to their excellent biocompatibility with a wide variety of cells and tissues, biodegradability, non‐toxicity, low immunogenicity, and microbial protection [14, 15, 16]. Common biopolymers used to produce these systems include alginate and gellan gum.

Recent research has studied drug delivery systems for improving diabetic wound repair, especially using natural polymers in advanced hydrogel formulations [1, 17]. Fan et al. [18] demonstrated that hydrogels of poly(vinyl alcohol), chitosan, and sodium alginate containing curcumin were efficient in accelerating diabetic wound healing, while Li et al. [19] described a multifunctional hydrogel of hyaluronic acid with antimicrobial peptide and micelles containing quercetin as a promising strategy for improving the repair of diabetic wounds. In another work, Abdel‐Gawad et al. [20] reported wet hydrogels of chitosan and collagen crosslinked with genipin and loaded with Ag nanoparticles. The results showed that the hydrogel was able to prevent microbial infiltration and release the antimicrobial payload, resulting in excellent wound healing. Roska et al. [21] developed whey protein microparticles containing chloramphenicol incorporated into the chitosan hydrogel system, promoting improved efficacy in the treatment of dermal wounds, demonstrating enhanced antimicrobial effects and promotion of healing. However, most of the previous research has focused on single drug systems.

For wound healing applications, dressings can be loaded with compounds that provide therapeutic benefits, including antimicrobial agents and pain relief medications [22]. Bupivacaine is a potent, long‐acting amide‐type local anesthetic [23], while gentamicin is an aminoglycoside antibiotic used to treat various bacterial infections [24]. The sustained release of gentamicin can enhance treatment efficacy and safety, minimizing the risk of adverse effects. Furthermore, microencapsulation of gentamicin can protect the drug from degradation [24, 25]. The strategy of combining anesthetic and antibiotic has already been explored by other authors but using dual drug delivery systems based on electrospun nanofibers [26, 27, 28]or membrane [29].

This study aimed to develop a dual‐drug delivery system composed exclusively of natural polymers using alginate microparticles embedded within a gellan gum/collagen hydrogel. Gentamicin was loaded into the microparticles, while bupivacaine was incorporated directly into the hydrogel, enabling the independent and controlled release of both drugs. The formulation was designed with potential applicability as a preformed, moist wound dressing, in alignment with recent developments in the field [30, 31, 32]. The system stands out for its simple preparation methodology and scalability.

## 2 Materials and Methods

### 2.1 Materials

The materials used to prepare the dual‐drug delivery systems were sodium alginate (GRINDSTED Alginate FD 175, 350–550 mPa.s for 1% solution, M/G ratio = 0.6, CAS 9005‐38‐3) kindly provided by Danisco (Denmark), highly acylated gellan gum (KELCOGEL CG‐HA, CAS 71010–52‐1) kindly supplied by CPKelco (USA), bovine collagen in powder (NOVAPROPÓ) kindly provided by NovaProm (Brazil) and calcium chloride P.A. (CAS 10043‐52‐4, Synth, Brazil). Ninhydrin (CAS 485–47‐2, ACS reagent), phosphate‐buffered saline pH 7.4, and the drugs bupivacaine hydrochloride monohydrate (CAS 73360‐54‐0, analytical standard ≥ 99%) and gentamicin sulfate (CAS 1405‐41‐0, USP testing specifications) were purchased from Sigma‐Aldrich (USA). All chemicals were of analytical grade.

### 2.2 Methods

#### 2.2.1 Preparation of Alginate Microparticles

**FIGURE 1:**
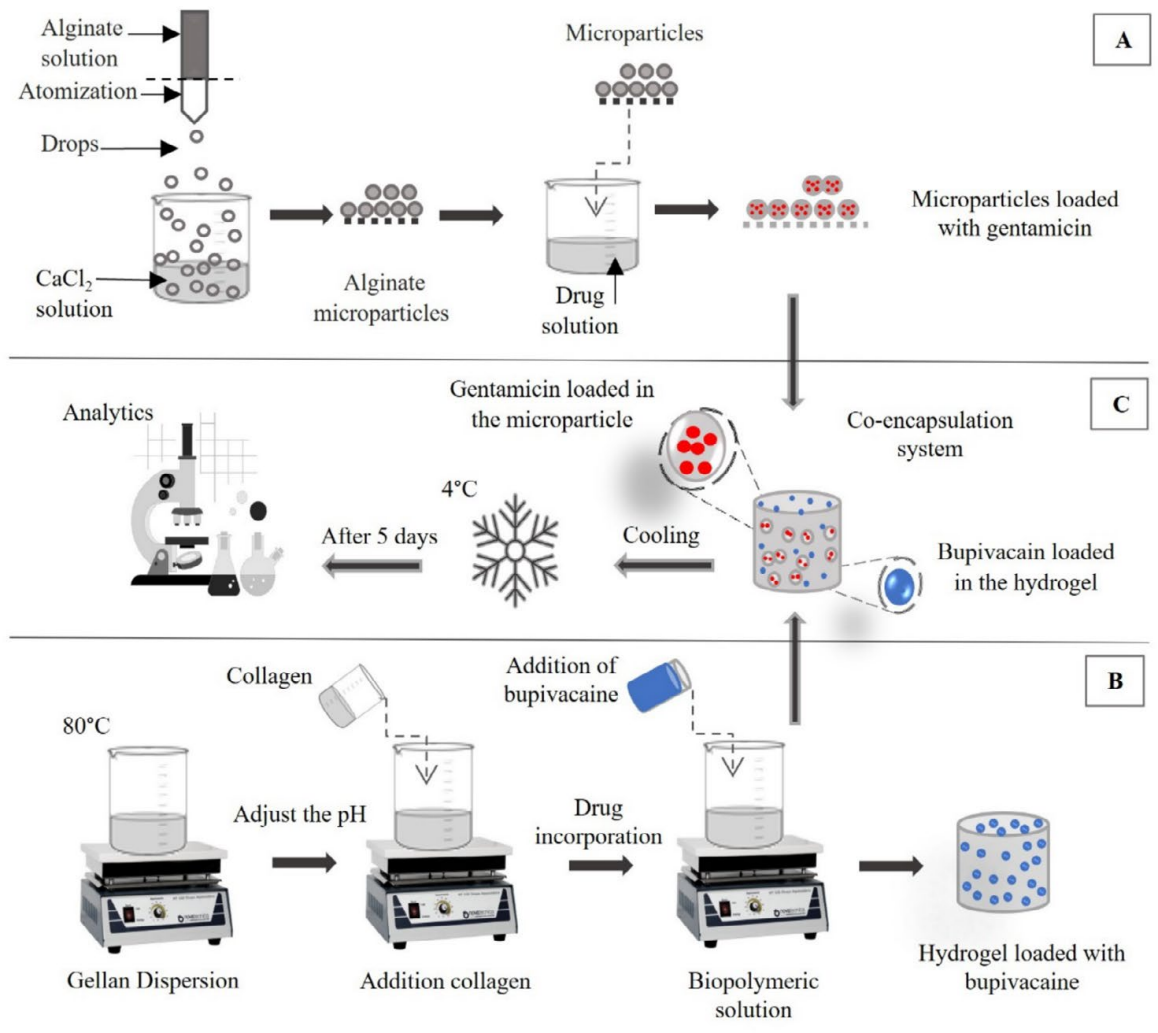
Schematic representation of the methodology used to prepare the dual‐drug delivery systems. (A) Preparation of alginate microparticles loaded with gentamicin, (B) preparation of gellan gum/collagen hydrogels loaded with bupivacaine, and (C) preparation of the dual‐drug delivery system.

A sodium alginate solution (3%, w/v) was prepared by gradually adding alginate powder to deionized water under magnetic stirring at room temperature until complete dissolution. To prepare the microparticles, the alginate solution was atomized into a calcium chloride solution (1%, w/v) under magnetic stirring. This process utilized an atomization system consisting of an atomizer nozzle with a 0.5 mm diameter (Labmaq, Brazil), a peristaltic pump TE‐BP‐01 Mini (Tecnal, Brazil), and a compressor model OP8.1/30II (Pressure, Brazil). Figure 1A shows a schematic representation of the procedure used to produce the alginate microparticles. The operational parameters included a distance of 10 cm between the nozzle and calcium chloride solution, a compressed air pressure of 0.8 bar, and a flow rate of approximately 0.045 L/h. The gelled particles were kept in the salt solution for 30 min, then filtered, and stored at 4°C.

#### 2.2.2 Incorporation of Gentamicin Into the Microparticles

Gentamicin was incorporated into microparticles through an adsorption process. The adsorption capacity of the microparticles was evaluated using the method described by Pássaro et al. [33]. For the adsorption, 1 mL of gentamicin solution (0.35%, w/v) was added to 0.1 g of wet microparticles, stirred at 30 rpm using a tube revolver (Thermo Scientific, USA) at room temperature (25°C), and evaluated at several time intervals (1, 3, 5, 10, 15, 30, 60, 180, 240, 270, 300, 330, 360, 390 and 420 min). Phases were separated by centrifugation at 4000 rpm (Centrifuge 5702, Eppendorf, Brazil), and the concentration of gentamicin in the solution was quantified using a UV–VIS spectrophotometer Genesys 10S (Thermo Scientific, EUA) at a wavelength of 401 nm. The adsorption capacity was calculated using Equation (1).

(1) $$q^{*}=\frac{V_{\mathrm{sol}}\cdot \left(C_{0}-C^{*}\right)}{m_{\mathrm{ads}}}$$

where q* is the adsorption capacity (mg of adsorbed drug/g adsorbent), *V*_*sol*_ is the volume of solution containing the drug to be adsorbed (1 mL), *C*_0_ and C* are the initial and the equilibrium gentamicin concentrations, respectively, and *m*_*ads*_ is the mass of adsorbent (g).

After determining the adsorption capacity, gentamicin was incorporated into the microparticles for subsequent experiments using a batch adsorption method, immersing 0.1 g of wet microparticles in 1 mL gentamicin solution (0.35% w/v) for 5 min (Figure 1A).

#### 2.2.3 Hydrogel Preparation

Hydrogels composed of gellan gum and collagen were prepared by mixing these biopolymers in a mass ratio of 95:5 (gellan gum: collagen) in deionized water, resulting in a total biopolymer concentration of 1% (w/v). The mixtures were heated to 80°C for 30 min, after which the pH was adjusted to 7.0. These conditions were established in preliminary experiments, and at this stage, the drugs were not incorporated into the systems. The mixtures were placed into cylindrical molds (17 mm in diameter and 30 mm in height), rapidly cooled to 4°C, and maintained at this temperature for 5 days until analysis (Figure 1B).

#### 2.2.4 Preparation of Dual‐Drug Delivery Systems

Dual‐drug delivery systems were prepared by incorporating gentamicin‐loaded microparticles and 0.01 g of bupivacaine into 4 mL of a gellan gum/collagen solution, after heat treatment at 80°C for 30 min. Systems containing 0, 0.02, 0.1, 0.2, 1, and 2 g of microparticles in 4 mL of hydrogel were evaluated, representing microparticle concentrations ranging from 0% to 50% (w/v) in the hydrogel. The resulting mixtures were transferred to cylindrical molds (17 mm in diameter and 30 mm inheight), rapidly cooled to 4°C, and maintained at this temperature until further analysis. A schematic diagram of the preparation process is presented in Figure 1B,C. The final product is designed to be a preformed, hydrated hydrogel, stored and used under moist conditions, similar to currently available commercial wound dressings.

#### 2.2.5 Characterization

The characterization of microparticles regarding size and shape and the characterization of hydrogels, with or without microparticles, related to morphology, mechanical, thermal, and chemical properties was performed in the absence of the drugs. The drugs gentamicin and bupivacaine were only added to the samples, as shown in Figure 1, for quantification of incorporation efficiency and drug release kinetics.

##### 2.2.5.1 Quantification of Gentamicin

Initially, the colorimetric reagent ninhydrin was prepared by dissolving 50 mg of ninhydrin in 10 mL of phosphate‐buffered saline (PBS) (pH 7.4) to obtain a stock solution with a concentration of 5 mg/mL. The stock solution was stored at 4°C and protected from light.

For the quantification of gentamicin, a complex of gentamicin and ninhydrin in PBS is formed, resulting in the development of a purple color in the solution. A wavelength scan was performed to select the optimal wavelength for quantifying the gentamicin‐ninhydrin complex without interference from other components. The wavelength of 401 nm [24] was chosen for the quantification of gentamicin, with PBS used as the blank.

To prepare the standard curve, a gentamicin stock solution was prepared by dissolving 1 g of gentamicin in 10 mL of PBS solution (pH 7.4), resulting in a final concentration of 100 mg/mL (10% w/v). This stock solution was subsequently diluted from 50 to 500 μg/mL and quantified to generate the standard curve (*y* = 0.0017 *x* + 0.015; *r* ^2^ = 0.9991) (Figure S1).

##### 2.2.5.2 Quantification of Bupivacaine

A stock solution with a concentration of 500 μg/mL was prepared by dissolving 5 mg of bupivacaine in 10 mL of phosphate‐buffered saline (PBS) (pH 7.4). The solution was mixed under magnetic stirring and placed in an ultrasound bath for 5 min. The solution was then diluted from 10 to 250 μg/mL (*y* = 0.0039*x*—0.0025; *r* ^2^ = 0.9996) to prepare the standard curve (Figure S2). The drug was quantified using a UV–VIS spectrophotometer with a wavelength of 263 nm [34].

##### 2.2.5.3 Size and Shape of Microparticles

The shape and size of the microparticles were evaluated by optical microscopy using a Primo Star optical microscope (Carl Zeiss, Germany) with a 40× objective. The particles were assessed by image analysis using the free software Image J (https://imagej.nih.gov/ij/). For this, about 400 particles from 5 images were measured. The particle size was determined as the average Sauter diameter (Equation (2)).

(2) $$d_{32}=\frac{\sum n_{i}d_{i}^{3}}{\sum {n_{i}d}_{i}^{2}}$$

where *n*_*i*_ is the number of microparticles with diameter *d*_*i*_.

For the characterization of microparticles shape, maximum (Fmax) and minimum (Fmin) Feret diameters were measured and the aspect ratio (AR) was determined by the ratio between them, as shown in Equation (3).

(3) $$\mathrm{AR}=\frac{\text{Fmax}}{\;\text{Fmin}}$$

##### 2.2.5.4 Incorporation Efficiency of Gentamicin in the Microparticles

The incorporation efficiency was determined by the ratio of the amount of drug incorporated in the microparticles to the amount of drug initially added, as described in Equation (4) [35]. These measurements were performed at time intervals ranging from 5 to 360 min during the adsorption of gentamicin into the microparticles. The amount of drug incorporated was calculated by mass balance, based on the amount of drug quantified in the supernatant at each time point during the adsorption process.

(4) $$\text{Incorporation efficiency}\left(\%\right)=\frac{\text{Mass of drug incorporated}}{\text{Mass of drug initially added}}\cdot 100\%$$

The quantification of gentamicin was performed using a UV–VIS spectrophotometer Genesys 10S (Thermo Scientific, USA) at a wavelength of 401 nm, as described in section 2.2.5.1. Each formulation was quantified in triplicate, and the mean values were used for the calculations.

##### 2.2.5.5 Stability of Microparticles in Aqueous Media

The stability test in aqueous media was conducted with 0.1 g of microparticles suspended in 10 mL of water or phosphate‐buffered saline at 37°C for 7 days. After 7 days, the samples were centrifuged (4000 rpm, 5 min), and the mass of the microparticles was determined.

##### 2.2.5.6 Scanning Electron Microscopy (SEM)

The morphology of microparticles and hydrogels (samples of approximately 5 mm x 5 mm x 3 mm) was analyzed using a scanning electron microscope (SEM). The samples were frozen in liquid nitrogen and freeze‐dried for 24 h to preserve their original structure. After freeze‐drying, the samples were fractured and covered with gold in the Sputter Coater EMITECH, Model K450 (Kent, UK), with an Au layer thickness of approximately 200 Å. The samples were then examined in a Scanning Electron Microscope model Leo 440i (LEO Electron Microscopy, England) at an acceleration voltage of 10 kV and a current of 50 mA.

##### 2.2.5.7 Mechanical Properties

The mechanical properties of hydrogels were evaluated by uniaxial compression using a CT3 Texture Analyzer texturometer (Brookfield Engineering, USA) with a cylindrical probe of 40 mm diameter. The mechanical properties at fracture were determined by compressing the gels (17 mm in diameter and 30 mm in height) to 90% of their initial height at a speed of 1 mm/s. Hencky stress (*σ*_H_) and Hencky strain (*ε*_H_) were calculated from force‐height data according to Equations (5) and (6), respectively.

(5) $$\sigma_{H}=F\left(t\right)\;\frac{H\left(t\right)\;}{H0\cdot A0\;}$$

(6) $$\varepsilon_{H}=\ln\;\left(\frac{\;H\left(t\right)\;}{H0}\right)$$

where *F*(*t*) is the force at time *t* (*N*), *A*_0_ is the initial area (m^2^), *H*_0_ is the initial height (m), and *H*(*t*) is the height at time *t* (m).

##### 2.2.5.8 Water Holding Capacity

To analyze the water holding capacity, approximately 0.8 g of hydrogel was placed on a Whatman # 1 filter paper disc (Whatman, UK), and positioned in centrifuge tubes. After centrifugation at 180×*g* for 10 min, the filter paper with the absorbed water was removed and weighed. The measurements were performed in triplicate at room temperature. The water holding capacity (WHC) was expressed as the percentage of water retained in the gel after centrifugation (Equation (7)).

(7) $$\mathrm{WHC}\;\left(\%\right)=100\;\left[1-\left(\frac{\;{\text{water}}_{\text{loss}}\;\left(g\right)\;}{\;{\text{water}}_{\mathrm{gel}\;}\;\left(g\right)}\right)\right]$$

where ${\text{water}}_{\text{loss}}$ is the amount of water released after centrifugation and ${\text{water}}_{\mathrm{gel}}$ is the initial amount of water.

##### 2.2.5.9 Oscillatory Rheology

The gelation process and the rheological properties of the samples were evaluated by oscillatory tests on an MCR92 rheometer (Anton Paar, Austria) equipped with a 50 mm diameter plate‐plate geometry and a 1 mm gap. The gellan gum/collagen solutions, with or without added microparticles, were transferred to the preheated rheometer plate (80°C) immediately after heat treatment. The samples were homogenized by pre‐shearing at 100 s^−1^ for 2 min and then allowed to rest for 5 min before measurements. Temperature sweeps were performed (cooling–heating–cooling) between 20°C and 80°C, with a heating/cooling rate of 1°C/min, at 0.1 Hz and 1% strain (within the linear viscoelastic range). The gel point was determined by the crossover between *G*′ and *G*″ [36].

##### 2.2.5.10 Fourier Transform Infrared Spectroscopy (FTIR)

The functional groups and possible interactions between the biopolymers were evaluated by Fourier transform infrared spectroscopy (FTIR) using an Agilent Cary 630 FTIR spectrometer (California, USA) in ATR mode. The spectra were obtained in the freeze‐dried samples, to avoid interference of water peaks, by averaging 16 scans between 400 and 4000 cm^−1^ with a resolution of 2 cm^−1^ [37, 38].

##### 2.2.5.11 Thermal Characterization

Thermal characterization of microparticles and hydrogels was performed by differential scanning calorimetry (DSC) and thermogravimetric analysis (TGA). The samples were frozen in liquid nitrogen and freeze‐dried for 24 h before analysis, which was carried out using a TGA/DSC1D instrument (Mettler Toledo, Switzerland). For DSC, the samples were heated from 25°C to 160°C with a heating rate of 10°C/min in an inert atmosphere of nitrogen (50 mL/min) [38]. For TGA, the samples were heated from 25°C to 600°C at a heating rate of 10°C/min. DTGA (first derivative of TGA) curves were used to better visualize the main peaks associated with weight loss.

#### 2.2.6 Drug Release Kinetics

For the release study, microparticles, hydrogels, and dual‐drug delivery systems containing the drugs were immersed in 50 mL of phosphate buffer solution (PBS) at 37°C under constant stirring in a Dubnoff thermostatic bath (Ethiktechnology, Brazil). Sink conditions were respected for both gentamicin and bupivacaine release. Table 1 provides the nomenclature of the systems evaluated for release kinetics, with systems containing 0.1 g of alginate microparticles corresponding to a concentration of 2.5% (w/v) of microparticles in the hydrogel. Aliquots of 1 mL were collected at predetermined time intervals, with partial medium renewal. Drug quantification was performed using a spectrophotometer at wavelengths of 401 nm and 263 nm, as described in Sections 2.2.5.1 and 2.2.5.2, respectively.

**TABLE 1:** Nomenclature of the systems that were evaluated by the release kinetics.

System	Description
MP(GEN)	0.1 g of alginate microparticles containing gentamicin
H(BPV)	4 mL of gellan gum/collagen hydrogel containing bupivacaine
H‐MP(GEN)	Dual‐drug delivery system: 4 mL of gellan gum/collagen hydrogel containing bupivacaine +0.1 g of alginate microparticles containing gentamicin—gentamicin release analysis
H(BPV)‐MP	Dual‐drug delivery system: 4 mL of gellan gum/collagen hydrogel containing bupivacaine +0.1 g of microparticles containing gentamicin—bupivacaine release analysis

From the kinetic data of the release tests, theoretical and empirical mathematical models were fitted to understand the behavior of the studied systems and to predict the mechanisms involved in drug release.

#### 2.2.7 Statistical Analysis

The analysis of variance (ANOVA) was performed using the Tukey test with Statistica software to assess significant differences at the 90% significance level (*p* < 0.1).

## 3 Results and Discussion

### 3.1 Alginate Microparticles Containing Gentamicin

**FIGURE 2:**
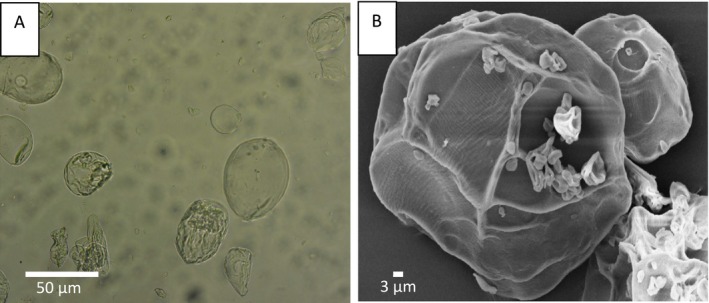
(A) Optical microscopy and (B) scanning electron microscopy micrographs of the alginate microparticles.

Figure 2 shows optical microscopy and scanning electron microscopy (SEM) images of alginate microparticles. The images reveal that the microparticles were irregularly shaped and individually dispersed, with a mean diameter of 34.97 ± 15.04 μm and an aspect ratio of 1.45 ± 0.28. The dispersion of the microparticles is crucial for their incorporation into hydrogels and makes them ideal for controlled‐release applications.

The size of the microparticles depends on the methodology and process conditions used in their preparation. In the atomization method, the alginate solution is pumped and atomized, with particle size controlled by the diameter of the atomizer nozzle, fluid flow rate, and compressed air flow rate [35]. The microparticles were similar to those presented by Rodríguez‐llimos et al. [39], who developed alginate microparticles containing paracetamol, which were irregular in shape and had a diameter ranging from 30 to 350 μm. Oliva‐Rodríguez et al. [40] observed alginate microparticles with diameters between 120 and 220 μm and an irregular structure, with the larger sizes attributed to particle formation by dripping, without the use of compressed air. Scanning electron microscopy (SEM) images (Figure 2B) showed that the microparticles had a rough surface, similar to those produced by the gelation/extrusion technique described by Tsirigotis‐Maniecka et al. [41].

Table 2 shows the results of the gentamicin adsorption into the alginate microparticles and the incorporation efficiency at each interval of the adsorption process. These results demonstrate the high incorporation efficiency of gentamicin into alginate microparticles at all the evaluated time points (> 86%). Ismail et al. [42] studied PLGA microparticles loaded with gentamicin and observed encapsulation efficiencies ranging from 11.64% to 42.98% for microparticles with diameters between 1.6 and 5.1 μm. Dorati et al. [24] reported encapsulation efficiencies between 43% and 64% for gentamicin‐loaded PEG‐PLGA nanoparticles with diameters ranging from 140 to 919 nm. These findings suggest that the gentamicin incorporation efficiency achieved for alginate microparticles is higher than the typical encapsulation efficiencies reported in the literature, underscoring the efficacy of the adsorption process in this system.

**TABLE 2:** Incorporation efficiency of gentamicin in alginate microparticles over time.

Time (min)	Gentamicin concentration (μg/mL)	Incorporation efficiency (%)
1	3300.78 ± 5.7	94.3 ± 0.05
3	3291.76 ± 6.0	94.1 ± 0.01
5	3325.69 ± 4.8	95.0 ± 0.01
10	3310.98 ± 0.9	94.6 ± 0.01
15	3288.82 ± 1.0	94.0 ± 0.01
30	3253.92 ± 1.9	93.0 ± 0.01
60	3174.90 ± 2.7	90.7 ± 0.01
180	3011.57 ± 5.3	86.0 ± 0.02
240	3008.43 ± 1.9	86.0 ± 0.01
360	3032.55 ± 4.3	86.6 ± 0.01

The highest incorporation efficiency (95%) was observed between 5 and 10 min, followed by a decrease in efficiency as the adsorption time increased (Table 2). Rapid drug adsorption likely occurred due to electrostatic interactions between gentamicin and the alginate microparticles. These attractive interactions are attributed to the zeta potential of alginate particles at pH 7.0, which is around −18 mV [43], while gentamicin is positively charged at pH 7.0, with a zeta potential of approximately +0.6 mV [25]. Additionally, the high superficial area of the microparticles may further favor the adsorption process.

The reduction in incorporation efficiency over longer periods (Table 2) may be directly related to the loss of mass from microparticles during the adsorption process. The stability of the microparticles was evaluated in aqueous media over 7 days, and a gradual mass loss was observed, with 86.1% mass loss in PBS solution and 26.3% in water. These differences in stability between the two media can be attributed to phosphate ions in PBS buffer, which act as calcium sequestrants, promoting particle destabilization [44]. A significant mass decrease after a few hours in a neutral aqueous medium was also reported by Tsirigotis‐Maniecka et al. [41] for alginate particles, which was attributed to the degradation and/or dissolution of the alginate matrix.

The adsorption capacity (q*) of alginate microparticles was estimated to be 32.8 mg/g, which is higher than that of similar gentamicin carriers reported in the literature. For example, Chen et al. [45] found a maximum adsorption capacity of 20.9 mg/g for (polyethylenimine)‐grafted bacterial cellulose, while Alvarez et al. [46] reported an adsorption capacity of less than 0.6 mg/g for silica nanoparticle–collagen composite hydrogels. These results highlight the strong adsorption ability of alginate microparticles for gentamicin.

Since the maximum incorporation efficiency of gentamicin into the microparticles was reached at 5 min, this time point was selected for the adsorption process in the production of the dual‐drug delivery systems.

### 3.2 Incorporation of Microparticles Into Hydrogels

#### 3.2.1 Morphology

**FIGURE 3:**
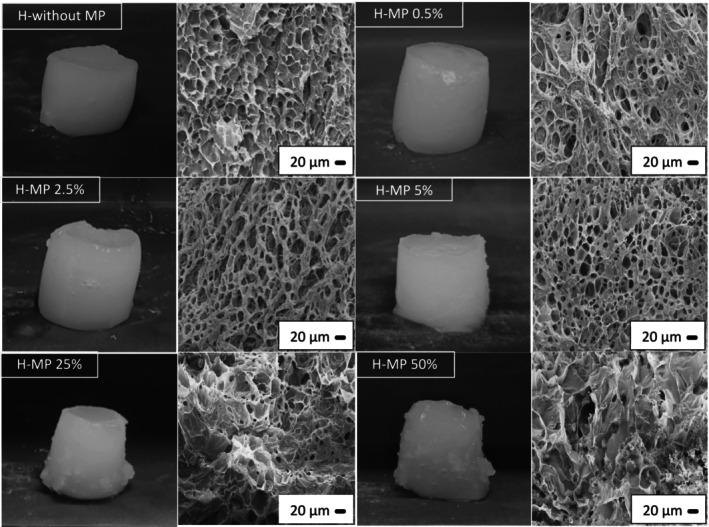
Photographs and SEM micrographs of gellan gum/collagen hydrogels without and with adding different concentrations of alginate microparticles (MP).

Figure 3 shows the photographs and SEM micrographs of the hydrogels prepared from gellan gum and collagen (95:5 gellan gum: collagen), with or without the addition of 0%, 0.5%, 2.5%, 5%, 25%, and 50% (w/v) alginate microparticles. The hydrogels were predominantly composed of gellan gum, which undergoes a two‐stage gelling process during cooling: first, the formation of double helices from a random coil, followed by the aggregation of helices into a continuous three‐dimensional network. The chain associations occur via physical crosslinking, through electrostatic forces and hydrogen bonds [47]. These hydrogels are resistant to dissolution in water.

All the hydrogels exhibited similar appearance characteristics. However, the addition of 25% and 50% (w/v) microparticles resulted in hydrogels with a more heterogeneous appearance, with microparticles present on the hydrogel surface.

SEM micrographs revealed an interconnected porous structure forming the hydrogel matrix. Similar microstructures were observed by Yamamoto et al. [47] for gellan gum gel, confirming that the hydrogel network was mainly composed of this polysaccharide. Hydrogels without microparticles and those with low microparticle concentrations (0.5%, 2.5%, and 5% w/v) exhibited similar structures. Microparticles were observed entrapped within the network, particularly in samples containing 25% and 50% w/v microparticles. These results confirmed the incorporation of microparticles (Figure S3), which remained intact after hydrogel preparation and were uniformly distributed throughout the structure. Furthermore, the addition of higher microparticle concentrations (25% and 50% w/v) led to the formation of more heterogeneous systems, with regions of a more compact network. Thus, the inclusion of high microparticle concentrations influenced the hydrogel microstructure, which may affect its macroscopic properties.

#### 3.2.2 Mechanical Properties and Water Holding Capacity (WHC)

Table 3 presents the mechanical properties of hydrogels with or without the addition of microparticles. The incorporation of lower amounts of microparticles (0.5% and 2.5% w/v) resulted in a reduction in stress at fracture and an increase in strain at fracture compared to hydrogels without microparticles (Table 3). Samples with intermediate microparticle concentrations (5% and 25% w/v) exhibited stress at fracture similar to hydrogels without microparticles, but their strain at fracture was higher, resembling that of systems with lower microparticle concentrations. However, adding higher amounts of microparticles (50% w/v) led to a significant increase in stress at fracture and a decrease in strain at fracture (Table 3). Stress at fracture can be used as an indicator of gel hardness, while strain at fracture reflects the elasticity of the gels [47]. Thus, adding lower amounts of microparticles resulted in less hard and more deformable hydrogels, whereas the highest microparticle concentration (50% w/v) produced harder and less deformable gels.

**TABLE 3:** Mechanical properties and water holding capacity of hydrogels with and without the addition of different amounts of microparticles.

Samples	Stress at fracture (kPa)	Strain at fracture (−)	Young's modulus (kPa)	WHC (%)
H‐without MP	11.26 ± 1.55ª^b^	0.373 ± 0.032^ab^	14.46 ± 1.49^b^	39.35 ± 0.02^a^
H‐MP 0.5%	8.82 ± 2.64ª	0.394 ± 0.071^ab^	15.55 ± 3.36^b^	29.50 ± 0.03^b^
H‐MP 2.5%	8.87 ± 1.24ª	0.431 ± 0.102^b^	9.18 ± 1.07^a^	40.31 ± 0.05^a^
H‐MP 5%	12.06 ± 1.32ª^b^	0.428 ± 0.118^b^	12.71 ± 3.18^ab^	41.06 ± 0.06^a^
H‐MP 25%	10.66 ± 1.74ª^b^	0.406 ± 0.073^b^	9.60 ± 2.28^a^	42.61 ± 0.07^a^
H‐MP 50%	14.95 ± 2.61^b^	0.263 ± 0.048^a^	16.03 ± 3.77^b^	45.26 ± 0.05^a^

*Note:* ^a–c^Equal letters on the same column indicate that data are not statistically different (*p* < 0.1).

The evaluation of mechanical properties (Table 3) provides insight into the effect of microparticle addition on the hydrogel structure. Young's modulus can be used to assess the interactions between the microparticles and the hydrogel network, according to van der Poel theory [48]. In the gels containing 2.5%–25% (w/v) microparticles, Young's modulus was lower than in the sample without microparticles, suggesting that the particles acted as inactive fillers and the interaction between the microparticles and the hydrogel network was not favored. In this case, the microparticles likely had the mobility to move within the hydrogel's porous structure during the compression test [49]. However, the observed reduction in stress at fracture suggests that the presence of microparticles affected the interaction between the gellan gum and collagen chains, leading to a decrease in the number of intramolecular bonds.

At the highest particle concentration (50% w/v), Young's modulus increased compared to the hydrogel without microparticles (Table 3), suggesting the influence of active particles [49]. In this case, reduced mobility leads to enhanced interactions between the particles and the biopolymeric network, resulting in the formation of a secondary microparticle network, which contributes to the reinforcement of the hydrogel [50]. This behavior is also consistent with the more compact structure at higher particle concentrations in the SEM micrographs (Figure 3).

The water holding capacity (WHC) results (Table 3) showed relatively low values (between ~29 and 45%), with a tendency for higher values in hydrogels containing a greater amount of microparticles. Picone and Cunha [36] reported water holding capacities above 60% for gellan gum gels. In contrast, Lee et al. [51] studied gellan gum/gelatin mixed gels and found that increasing the gelatin concentration (i.e., a low gellan:gelatin ratio) reduced WHC to around 40%. The highest WHC values were observed in hydrogels with higher microparticle concentrations (Table 3), which may be related to the smaller pore sizes in the hydrogel network (Figure 3) or to the ability of microparticles to retain water within the hydrogel matrix. This property is particularly relevant for the development of dressings, as a moist wound environment reduces pain, stimulates collagen synthesis, and supports cellular functions, ultimately leading to faster and higher‐quality healing [30].

#### 3.2.3 Oscillatory Rheological Analysis

**FIGURE 4:**
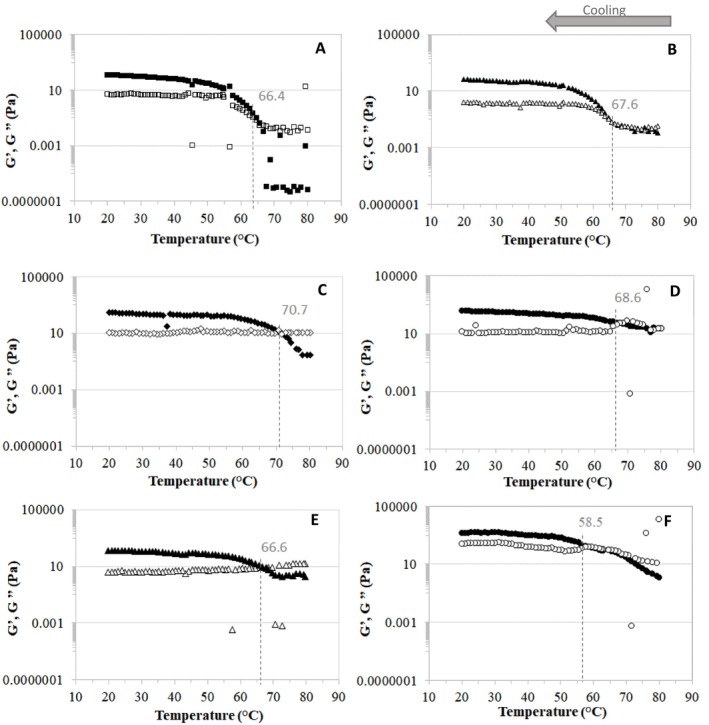
Elastic (*G*′) and viscous (*G*″) moduli and the crossover point of formation of hydrogels upon cooling gellan gum/collagen solutions with and without alginate microparticles. (A) Hydrogel without microparticle, (B) 0.5%, (C) 2.5%, (D) 5%, (E) 25% and (F) 50% w/v microparticles added in the hydrogel. Filled symbols represent *G*′ and open symbols represent *G*″.

Figure 4 shows the elastic modulus (*G*′) and viscous modulus (*G*″) upon cooling the gellan gum/collagen solutions with and without the addition of microparticles. The results indicate that *G*″ was greater than *G*′ at high temperatures, suggesting predominantly viscous behavior. The data exhibited a strong temperature dependence, with *G*′ increasing progressively as the temperature decreased, reaching higher values at lower temperatures due to gelation.

The temperature at which the *G*′ and *G*″ curves crossover, representing the gel point [52, 53], ranged from 50°C to 70°C. For hydrogel without microparticles, the crossover occurred at 66.4°C, and this temperature increased with higher microparticle concentrations. Additionally, the *G*′ curve exhibited plateaus before and after gelation, with the difference between these plateaus decreasing as the microparticle concentration increased. These results suggest that the addition of microparticles makes the biopolymer solution more elastic at higher temperatures. Furthermore, the data of the heating process (Figure S4) revealed that hydrogels with higher microparticle concentrations exhibit greater thermal stability. During heating, these hydrogels maintained higher complex viscosity values and displayed a more gradual decrease in ƞ*, indicating a slower structural breakdown. This behavior suggests that microparticles reinforce the gel network, delaying reversible gelation at higher temperatures, thereby stabilizing the hydrogel structure against thermal stress (Figure S4).

The *G*′ plateau after gelation was similar for all samples, except for the hydrogel containing 50% w/v microparticles, which exhibited higher *G*′ values. This result corroborates the findings of Young's modulus obtained under uniaxial compression (Table 3), suggesting that at low concentrations, the microparticles acted as inactive fillers, while at the highest concentration, they acted as active particles. Tomoda et al. [54] studied the incorporation of silk fibroin microparticles into fibroin hydrogels and observed a significant decrease in *G*′ with microparticle addition, which did not occur in the present work.

Systems containing 50% w/v microparticles exhibited the lowest gelation temperature (58.5°C) and the highest *G*′ values across the entire curve. These results support the hypothesis that a secondary network of microparticles is formed, or that interactions between the microparticles and the hydrogel network occur. This interaction likely reinforces the biopolymeric network, but it may also delay the gelation process of the gellan/collagen solution.

#### 3.2.4 Fourier Transform Infrared Spectroscopy (FTIR)

**FIGURE 5:**
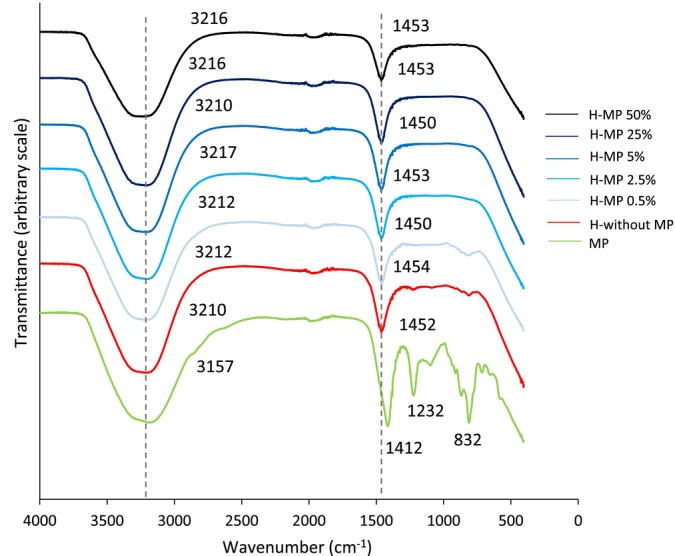
Infrared spectra of microparticles and hydrogels without and with adding microparticles.

Figure 5 shows the infrared spectra of microparticles and hydrogels, with or without the addition of microparticles. Broad bands between 3157 and 3217 cm^−1^ were observed, corresponding to the stretching vibration of OH groups in gellan gum. The hydrogels also exhibited a band around 1450 cm^−1^, which is attributed to the C—C aromatic stretching vibration modes of gellan gum [42].

The alginate microparticles exhibited vibrations at 1412 cm^−1^, corresponding to COO— stretching, at 1232 cm^−1^, associated with C—O stretching, and a peak at 832 cm^−1^ linked to mannuronic acid residues [55]. These results indicate that the intensity of the alginate bands decreased after incorporating the microparticles in the hydrogels, remaining only the characteristic bands of the gellan gum, the main component of the hydrogel. Furthermore, no new peaks appeared in the spectra of hydrogels containing microparticles, suggesting that strong covalent interactions were not formed between alginate (microparticles) and gellan gum/collagen (hydrogels). This supports the hypothesis that microparticles are physically incorporated into the hydrogel network.

#### 3.2.5 Thermal Analysis

**FIGURE 6:**
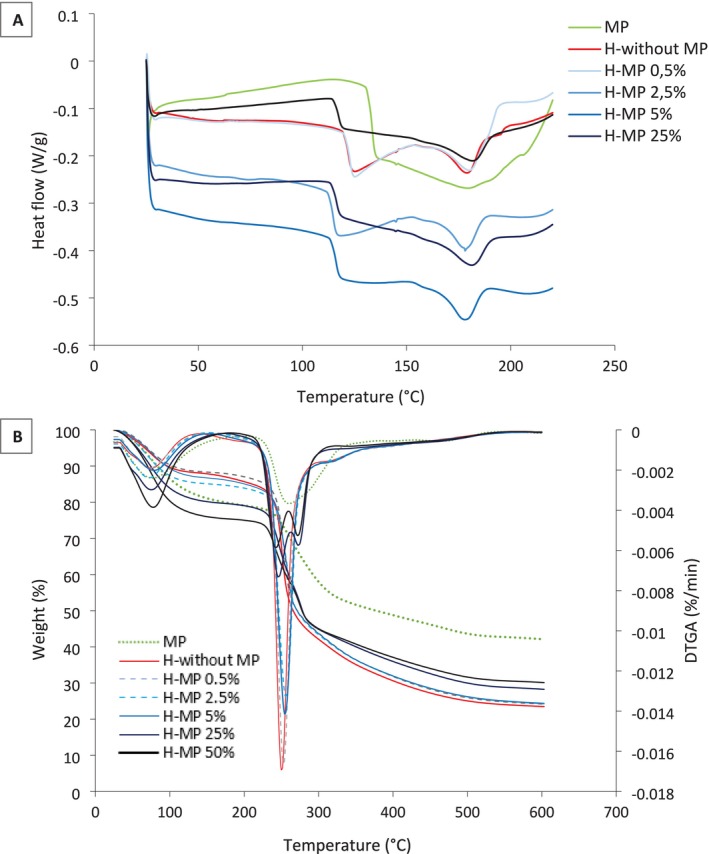
(A) Thermograms obtained by differential calorimetry (DSC) and (B) TGA and DTGA thermograms of microparticles and gellan gum/collagen hydrogels without and with adding alginate microparticles.

Differential Scanning Calorimetry (DSC) was performed to investigate the thermal behavior of sodium alginate microparticles and gellan gum/collagen hydrogels with and without microparticles (drug‐free formulations). The corresponding thermograms are shown in Figure 6A. The thermograms revealed two main endothermic events: the first event occurred between 119°C and 135°C, depending on the sample composition, and a second transition occurred at approximately 180°C.

The first event, observed between 119°C and 135°C, is associated with the melting transitions of the biopolymeric matrices. This peak was detected at 135°C for microparticles alone, while hydrogels containing 2.5%–50% w/v microparticles showed this thermal transition around 119°C. Hydrogels with 0.5% w/v microparticles and those without microparticles exhibited the melting transition closer to 125°C. These values align with previous findings, where gellan gum scaffolds displayed a melting point at 114°C [56] and nanoparticles of alginate at 119.7°C [57], supporting the identification of this transition as polymer melting. The first event cannot be attributed to a classical glass transition (*T*g), since *T*g values of dry polysaccharides such as gellan gum and alginate are generally observed below 100°C [58, 59]. A second endothermic transition, observed around 180°C in the hydrogels, can be attributed to the unfolding of the collagen triple helix into a random coil conformation [60].

Altogether, DSC results confirm that microparticle incorporation alters the thermal response of the hydrogels, shifting endothermic events and suggesting structural reorganization of the matrix. Importantly, these DSC findings are consistent with the TGA results, which showed polymer degradation only at higher temperatures (> 250°C), thereby supporting the interpretation that the transitions at 180°C are not related to gellan decomposition but rather to collagen denaturation or less stable domains within the hybrid composite [61].

Figure 6B shows the thermogram obtained from thermogravimetric analysis (TGA) and the DTGA derivative of alginate microparticles and gellan gum/collagen hydrogels, both with and without the addition of microparticles. TGA results indicate that both microparticles and hydrogels maintain thermal stability up to approximately 240°C. Two distinct thermal events are observed: the first around 100°C and the second near 250°C. The first event corresponds to water loss in all samples, while the second is associated with the degradation of the biopolymers. Yang et al. [62] reported a peak at 256°C, corresponding to the degradation temperature of gellan gum, while Durga et al. [63] observed a peak at 299°C for pure collagen. The presence of a single degradation peak in the second thermal event for the hydrogel without microparticles can be attributed to the higher concentration of gellan gum, which dominates the thermal properties of the hydrogels.

The thermogram for alginate microparticles also showed a first thermal event corresponding to water loss. In contrast, the second thermal event occurred over a broad range, from 215°C to 350°C, and is attributed to the degradation of alginate and the formation of carbonaceous residues [64].

The addition of a low concentration of microparticles did not affect the thermal behavior of the hydrogels. However, hydrogels with higher microparticle concentrations (25% and 50% w/v) exhibited two degradation peaks: one at 247°C and another at 264°C. The first peak is likely associated with the degradation of gellan gum, present in the hydrogel, while the second peak corresponds to the degradation of alginate, present in the microparticles. Interestingly, the second degradation peak, associated with the alginate microparticles, is slightly shifted to a higher temperature, supporting the hypothesis discussed in the mechanical and rheological tests that at higher concentrations, the microparticles behave as active fillers, forming a secondary network within the hydrogel. These interactions between the microparticles and the hydrogel matrix may contribute positively to formulation stability during storage and handling, even though the hydrogel is intended for moist, non‐thermal applications.

#### 3.2.6 Kinetics of Dual‐Drug Release and Mathematical Modeling

To produce the dual‐drug delivery systems, bupivacaine and gentamicin‐loaded microparticles were incorporated into the hydrogel matrix. It was not possible to load both drugs simultaneously into the hydrogel matrix without using microparticles, since a self‐sustaining structure was not formed (Figure S5). Both gentamicin and bupivacaine are positively charged at neutral pH and can electrostatically interact with gellan gum and collagen, which are negatively charged (Figure S6). However, bupivacaine has low solubility in aqueous media, limiting its interaction with the biopolymers [65]. In contrast, gentamicin is highly soluble [24] and can form insoluble complexes with the biopolymers or disrupt the interaction between gellan gum and collagen, potentially hindering gel formation. In this context, the gentamicin encapsulation in alginate microparticles allowed the incorporation of both drugs in the co‐encapsulation system without disrupting the hydrogel structure. Thus, the proposed dual‐delivery system was essential for enabling the simultaneous delivery of both gentamicin and bupivacaine from the same system.

**FIGURE 7:**
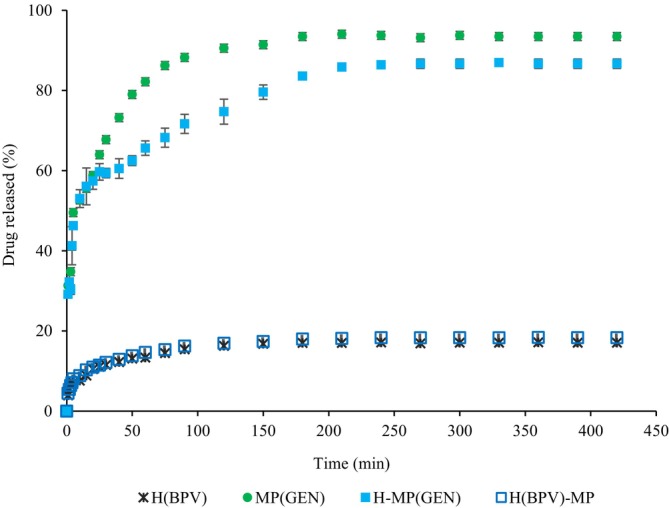
Kinetic curves for the release of gentamicin from microparticles (MP(GEN)) and dual‐drug delivery system (H‐MP(GEN)), and the release of bupivacaine from hydrogels (H(BPV)) and dual‐drug delivery system (H(BPV)‐MP).

The release profiles of bupivacaine and gentamicin incorporated into the hydrogel, microparticles, and dual‐drug delivery systems are shown in Figure 7. The release of both compounds reached equilibrium at approximately 200 min. For gentamicin release, the systems containing 2.5% (w/v) microparticles were evaluated. The incorporation of microparticles into the hydrogels (H‐MP(GEN)) increased resistance to diffusion, resulting in a slower and more controlled release of gentamicin compared to the free microparticles (MP(GEN)). These results demonstrate the efficacy of the dual‐drug delivery system in controlling the release profile. The mechanical properties of hydrogels, which were influenced by the incorporation of microparticles, may also affect drug release behavior. Increased stiffness or crosslink density can restrict molecular diffusion through the polymeric matrix, leading to slower drug release rates. Previous studies have demonstrated that more rigid hydrogel networks tend to delay the diffusion of both hydrophilic and hydrophobic compounds due to reduced pore size and water mobility [66, 67, 68]. Although a direct correlation was not quantitatively assessed in this study, the observed variations in mechanical strength and release profiles suggest a structure–function relationship that may be explored in future work.

As shown in Figure 7, 93% of gentamicin was released from the free alginate microparticles (MP(GEN)), while the release decreased to 86% in the dual‐drug delivery system (H‐MP(GEN)). The rapid initial release can be attributed to the higher concentration gradient and the strong affinity between gentamicin and the aqueous medium. Similar results were reported by Dorati et al. [24], who studied gentamicin‐loaded PEG‐PLGA/PLGA‐H nanoparticles and observed 70% release after 120 min, with complete release achieved after 600 min.

For bupivacaine, the release profile of hydrogels with and without microparticles (H(BPV)‐MP and H(BPV), respectively) was similar, with a maximum release of approximately 20% (Figure 7). The low release of bupivacaine may be attributed to the hydrophobic character of the drug, which limits its release into an aqueous medium. Additionally, bupivacaine has a pKa of 8.2 [65], favoring electrostatic interactions with gellan gum and collagen, both of which are negatively charged at neutral pH, thereby restraining its release into the medium. Notably, the slow release of bupivacaine could be beneficial, promoting prolonged and effective pain relief.

In this regard, the developed dual‐drug delivery system is promising, as it enables the simultaneous and independent release of an antibiotic (gentamicin) and an analgesic (bupivacaine), thereby optimizing both wound treatment and therapeutic action of the drugs.

**FIGURE 8:**
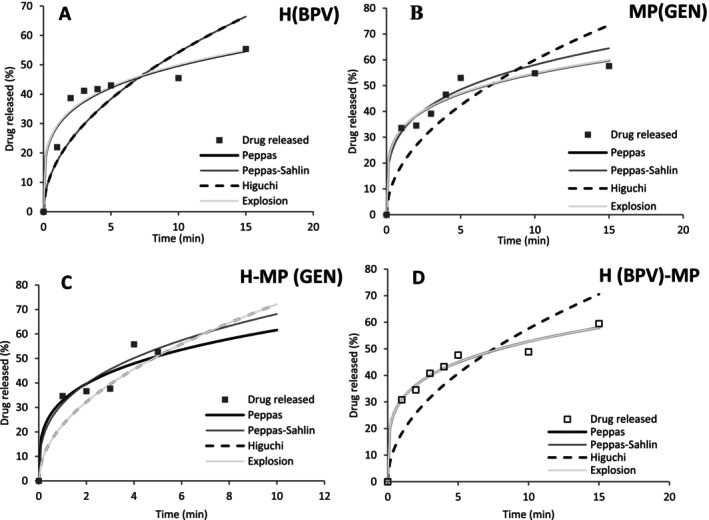
Fitting of mathematical models to the kinetic data of the release of drugs present in (A) hydrogels, (B) microparticles, (C, D) dual‐drug delivery systems.

Mathematical models were fitted to the kinetic data considering the restriction of the fitting up to 60% of the total released mass (𝑀𝑡/𝑀∞ ≤ 0.6) [69], as shown in Figure 8. The kinetic data were normalized based on the total mass released, and the experimental models of Peppas, Peppas‐Sahlin,and Higuchi were applied, along with a simplified version of the Explosion model for short times. The parameters obtained from fitting the different models are summarized in Table 4. The models showed a good fit to the kinetic data, as confirmed by cthe orrelation coefficient (*R* ^2^) greater than 88%, except for the Higuchi model. The Higuchi model was not suitable for representing the data, with R^2^ ranging from 67% to 82%.

**TABLE 4:** Fitting parameters of the models to the kinetic data of release.

		H(BPV)	MP(GEN)	H‐MP	
				H(BPV)‐MP	H‐MP(GEN)
Peppas $\frac{M_{t}}{M_{\infty }}={\mathrm{kt}}^{n}$	*k* (1/*s* ^*n*^)	29.066	32.941	30.966	32.815
	*n*	0.234	0.220	0.231	0.274
	*R* ^2^	0.941	0.967	0.985	0.939
Peppas‐Sahlin $\frac{M_{t}}{M_{\infty }}=k_{1}t^{m}+k_{2}t^{2m}$	*k*_1_ (1/*s* ^*m*^)	28.968	31.928	31.203	31.535
	*k*_2_ (1/*s* ^2*m*^)	0.000	0.000	0.027	0.000
	*m*	0.238	0.259	0.222	0.335
	*R* ^2^	0.929	0.939	0.982	0.887
Higuchi $\frac{M_{t}}{M_{\infty }}={\mathrm{kt}}^{1/2}$	*k* (1/*s* ^0.5^)	17.154	18.955	18.212	22.811
	*R* ^2^	0.692	0.677	0.722	0.827
	*K* (1/*s* ^*n*^)	29.231	33.006	30.966	32.842
Explosion $\frac{M_{t}}{M_{\infty }}={\mathrm{kt}}^{n}+b$	*n*	0.233	0.219	0.231	0.273
	*b*	0.000	0.000	0.000	0.000
	*R* ^2^	0.929	0.961	0.982	0.924

The Peppas model, widely used to describe drug release kinetics, presented the best fit to the experimental release data. The *R* ^2^ values found ranged from 94% to 98%, indicating an excellent correlation between the experimental data and the theoretical model. The Peppas parameter “*n*” is related to the drug release mechanism and may vary depending on the characteristics of the system [70]. From the fitting of the experimental data, the value of n was found between 0.22 and 0.27, which indicates a Fickian diffusion behavior in polydisperse systems [71]. In polydisperse systems, the particles present in the matrix have different sizes and characteristics, which can influence the release dynamics. The heterogeneity of the system can result in variations in the diffusion rates and drug release profiles, making diffusion models an essential tool for the characterization of these systems [49, 71].

A similar behavior was reported by Shoaib et al. [72], who observed *n* = 0.24 in studies involving the release of ibuprofen from HPMC matrices. This suggests that the analysis of n values can be a useful tool to better understand the release behavior of substances in controlled release systems. For the Peppas–Sahlin model, the value of the constant *k*_2_ was equal to zero, which implies that the constant *k*_1_ predominates in describing the behavior of the system, implying that the drug release mechanism is essentially controlled by diffusion rather than erosion or solubilization [49]. These results indicate that diffusion is the primary mechanism governing drug release, with minimal contribution from polymer relaxation or swelling.

The release of bupivacaine was studied in H(BPV)‐MP and H(BPV) systems, with the parameter n obtained for both systems being equal to 0.23, indicating that the release mechanism follows a characteristic diffusion behavior of the process predominantly governed by Fickian diffusion. The *R* ^2^ values for the Peppas, Peppas‐Sahlin and Explosion models varied between 92% and 98%, as shown in Table 4. These results suggest that the addition of microparticles to the hydrogel did not cause significant changes in the release kinetics of bupivacaine, indicating that the drug release mechanism remained predominantly unchanged, even with the incorporation of microparticles into the system. The absence of significant variations in the release rate suggests that the presence of microparticles does not substantially interfere in the diffusion processes or in the control of drug release from the hydrogel.

By comparison between the dual drug delivery systems H‐MP with H(BPV) and MP(GEN), it was possible to observe that the developed system was effective in the simultaneous release of gentamicin and bupivacaine. The release of gentamicin was significantly slower in the dual drug delivery system, compared to the free microparticles. This behavior suggests that the presence of the incorporation of the microparticles within the hydrogel system can alter the kinetics of gentamicin release, possibly due to the physical barrier imposed by the hydrogel itself. On the other hand, the release of bupivacaine from the hydrogels was not influenced by the incorporation of microparticles loaded with gentamicin. These findings demonstrate that, despite the presence of gentamicin, the release of bupivacaine was not impaired, evidencing the stability and efficacy of the system developed for combined drug delivery. These results are crucial for the development of more efficient controlled release systems, allowing the optimization of pharmaceutical formulations for more precise and controlled drug release.

## 4 Conclusion

Dual‐drug release systems were developed by incorporating alginate microparticles into gellan gum/collagen hydrogels. The microparticles had a mean diameter of 34.97 ± 15.04 μm, and the adsorption process for incorporating gentamicin was highly effective, achieving an incorporation efficiency of approximately 95%. The mechanical properties of the hydrogels were influenced by the addition of microparticles, likely due to changes in the hydrogel network structure. This change also affected the water‐holding capacity and microstructure, especially at the high microparticle concentrations. The dual‐drug delivery system successfully incorporated gentamicin and bupivacaine and resulted in slower gentamicin release compared to free microparticles. The release of bupivacaine was not influenced by the presence of gentamicin‐loaded microparticles but was slower than gentamicin release, likely due to the hydrophobic nature of bupivacaine. Overall, the developed dual‐drug delivery system demonstrates the ability to independently and sustainably release both bupivacaine and gentamicin, highlighting its potential as a prolonged‐release wound dressing that combines antimicrobial and analgesic effects.

## Conflicts of Interest

The authors declare no conflicts of interest.

## Supporting information


**Figure S1:** Calibration curve of gentamicin solution.
**Figure S2:** Calibration curve of bupivacaine solution.
**Figure S4:** Complex viscosity | ƞ*| of hydrogels with and without addition of sodium alginate microparticles. (a) cooling cycle, (b) heating cycle.
**Figure S5:** Appearance of hydrogel of gellan gum and collagen containing gentamicin and bupivacaine. (A) Hydrogel in the cylindrical mold and (B) hydrogel after demolding.
**Figure S6:** Zeta potential of gellan gum, collagen and sodium alginate solutions at different pH values.

## Data Availability

The data that support the findings of this study are available from the corresponding author upon reasonable request.
