# Supplementary material for: Advanced Biomaterial for Dual‐Drug Release: A Hydrogel‐Microparticle Approach
Source: Biopolymers. 2025 Sep 13;116(5):e70049. doi: 10.1002/bip.70049 (PMC12433186; doi:10.1002/bip.70049)
Supplement: Supplementary file 1 — Figure S1: Calibration curve of gentamicin solution. Figure S2: Calibration curve of bupivacaine solution. Figure S4: Complex viscosity | ƞ*| of hydrogels with and without addition of sodium alginate microparticles. (a) cooling cycle, (b) heating cycle. Figure S5: Appearance of hydrogel of gellan gum and collagen containing gentamicin and bupivacaine. (A) Hydrogel in the cylindrical mold and (B) hydrogel after demolding. Figure S6: Zeta potential of gellan gum, collagen and sodium alginate solutions at different pH values. [file BIP-116-e70049-s001.docx]

Supporting information for:

# Advanced Biomaterial for Dual-Drug Release: A Hydrogel-Microparticle Approach

## Jose Gregorio Fontainez Garrido^1^, Newton Andreo Filho^2^, Fabiana Perrechil^1^, Mariana Agostini de Moraes^1,3*^

## ^1^ Department of Chemical Engineering, Federal University of São Paulo - UNIFESP, Diadema, 09913-030, Brazil.

## ^2^ Department of Pharmaceutical Science, Federal University of São Paulo - UNIFESP, Diadema, 09913-030, Brazil.

## ^3^ School of Chemical Engineering, University of Campinas – UNICAMP, Campinas, 13083-852, Brazil.

*Corresponding author: agostini@unicamp.br

**B**

Figure S1. Calibration curve of gentamicin solution.

Figure S2 - Calibration curve of bupivacaine solution.

Figure S3. Micrograph of hydrogel fracture, showing the incorporation of microparticles.


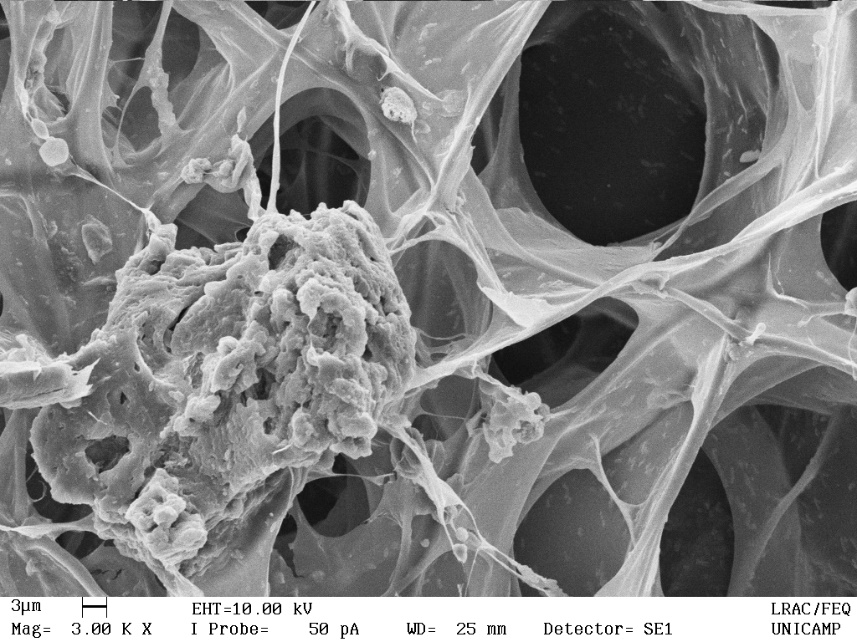


3 µm

H-MP 2,5%

**A**

A

B

**A**

Figure S4. Complex viscosity | ƞ*| of hydrogels with and without addition of sodium alginate microparticles. a) cooling cycle, b) heating cycle.


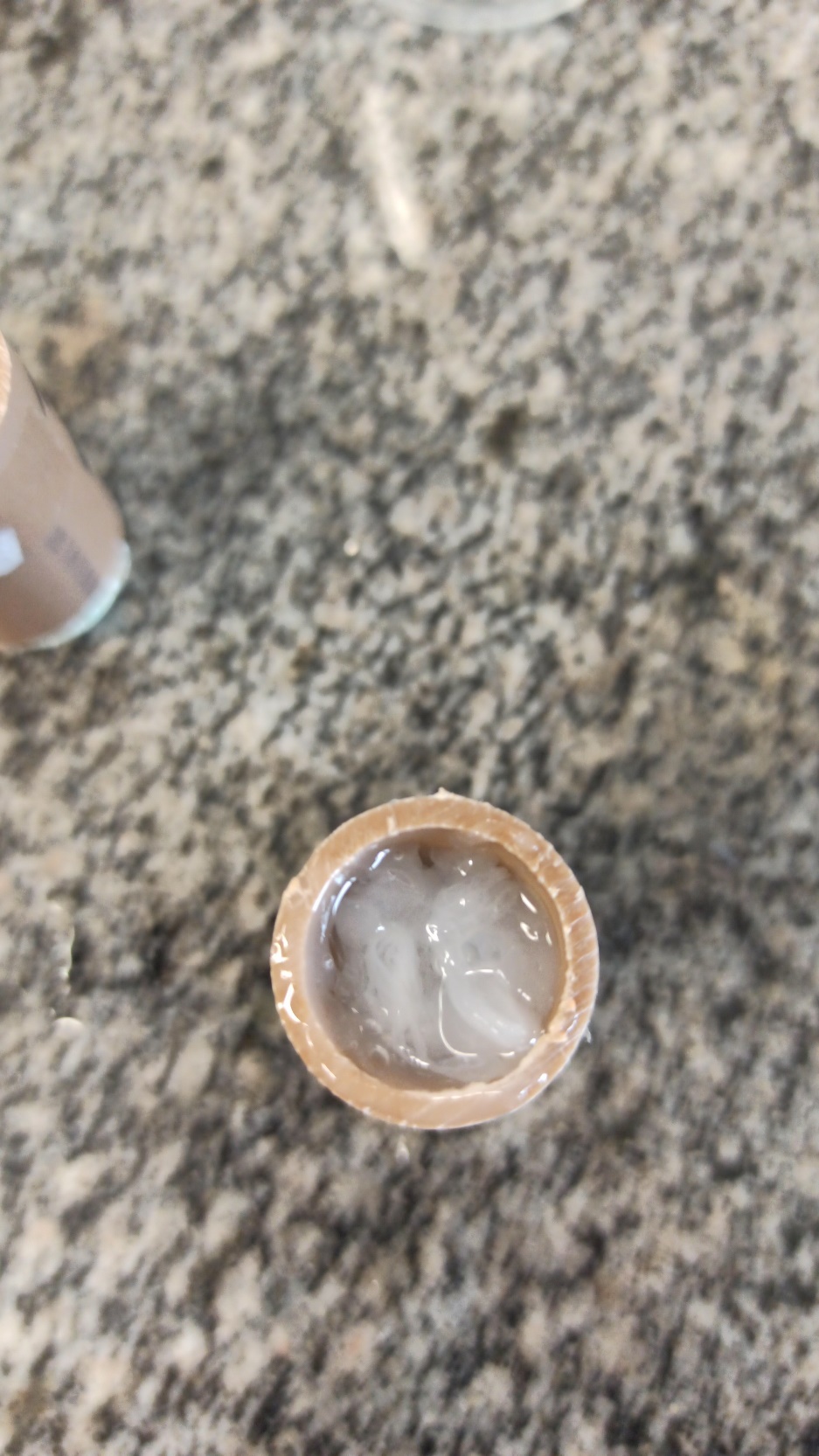

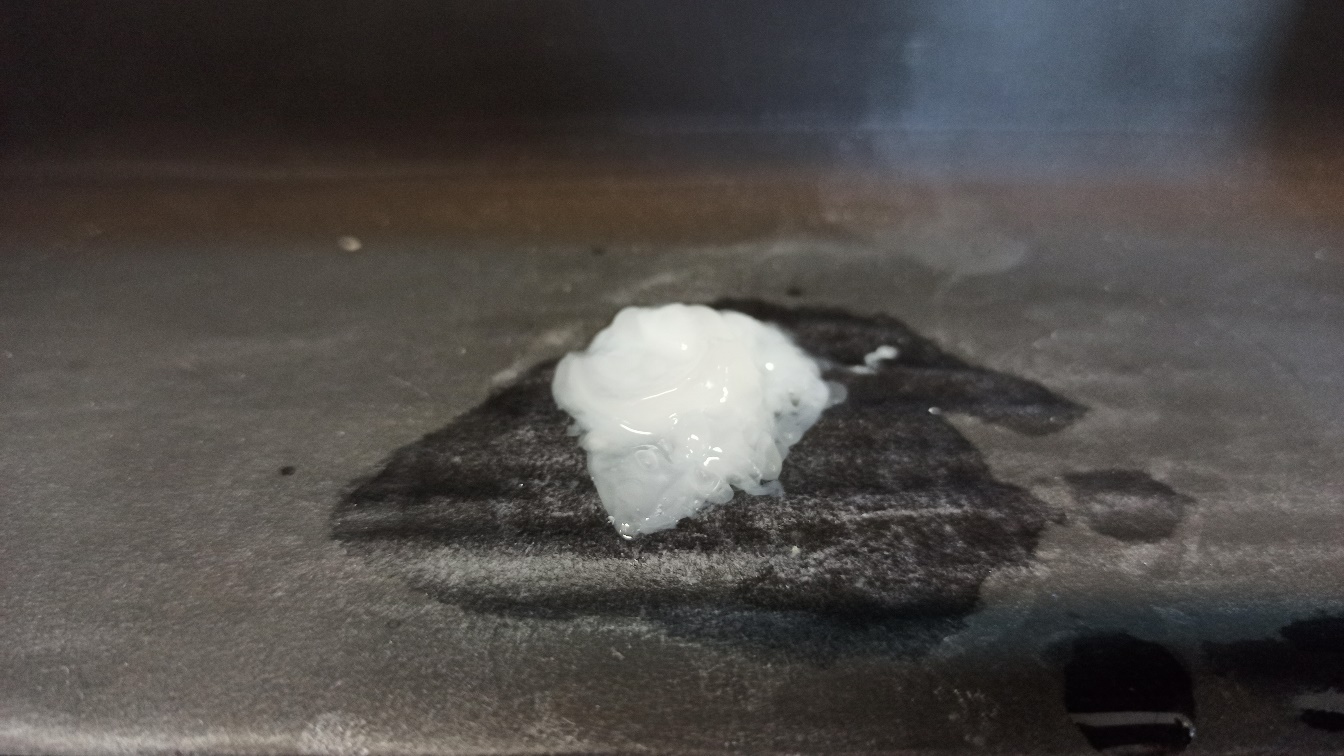


Figure S5. Appearance of hydrogel of gellan gum and collagen containing gentamicin and bupivacaine. A) Hydrogel in the cylindrical mold and, B) hydrogel after demolding.

Figure S6. Zeta potential of gellan gum, collagen and sodium alginate solutions at different pH values.
